# Geographic, Racial, and Sex Disparities in Time to Treatment for Early-Onset Colorectal Cancer

**DOI:** 10.1001/jamanetworkopen.2026.1980

**Published:** 2026-03-16

**Authors:** Meng-Han Tsai, Steven S. Coughlin, Jorge Cortes, Kenneth J. Vega

**Affiliations:** 1 Georgia Prevention Institute, Augusta University, Augusta; 2 Georgia Cancer Center, Augusta University, Augusta; 3 Department of Biostatistics, Data Science and Epidemiology, School of Public Health, Augusta University, Georgia; 4 Division of Gastroenterology and Hepatology, Prisma HealthMidlands, Columbia, South Carolina

## Abstract

This cross-sectional study investigates diagnostic delays and disparities—particularly those related to geography, race, and sex—in time to treatment for early-onset colorectal cancer.

## Introduction

Adults with early-onset colorectal cancer (EOCRC) often face diagnostic delays and advanced stage diagnosis, making timely treatment essential.^1,2^ Male patients and those from racially and/or ethnically minoritized groups, especially those in disadvantaged areas, are more likely to experience treatment delays.^3^ Limited studies have examined how sex, racial and ethnic, and geographic disparities impact treatment timeliness. This study addresses that critical gap by examining treatment timeliness across 3 postdiagnosis intervals. We hypothesized that rural patients would experience longer treatment delays, particularly among certain subgroups.

## Methods

Data extracted for this cross-sectional study were publicly available and deidentified and thus considered exempt from review by the institutional review board at Augusta University, and informed consent was not required. This study followed the STROBE reporting guidelines.

We conducted a retrospective analysis using the 2006 to 2020 incidence data from the Surveillance, Epidemiology, and End Results (SEER) Program. The exposures included sex, race, and census tract level rurality. Time to treatment (TTT) was categorized as initiation within 30, 60, or 90 days after diagnosis and was censored if treatment was not initiated (eMethods in Supplement 1). Bivariate differences were tested with χ^2^ tests, and adjusted associations were tested with Cox proportional hazards models. Both unadjusted models (including the 3 exposures only) and adjusted models were applied. Multiple imputation addressed missing treatment time data (11 312 patients [14.3%]). False discovery–rate (FDR) adjustment was applied to the multivariable analyses to account for multiple comparisons. Data were analyzed using SAS version 9.4 (SAS Institute), with statistical significance set at a 2-sided FDR-adjusted *P* < .05.

## Results

Among 79 090 patients with EOCRC (42 092 male [53.22%]; 58 316 individuals aged 40-49 years [73.9%]), the mean (SD) TTT was 20.0 (32.4) days. The mean (SD) TTT was shortest in mostly rural areas (17.8 [27.7] days) and longest in all urban areas (20.7 [33.2] days). Many male patients resided in all rural areas, while a greater proportion of female patients were in all-urban areas. Racially and ethnically minoritized groups predominantly lived in all-urban settings (Table 1). The imputed or adjusted model with stratified analyses revealed that male patients in all-urban areas were about 5% less likely to initiate treatment. Asian or Pacific Islander (hazard ratio [HR], 0.96; 95% CI, 0.93-0.99; FDR-adjusted *P* = .01), Black (HR, 0.95; 95% CI, 0.92-0.98; FDR-adjusted *P* = .001), and Hispanic (HR. 0.93; 95% CI, 0.91-0.95; FDR-adjusted *P* < .001) patients in all-urban areas were less likely to receive treatment within 90 days, with similar patterns observed at 30 and 60 days (Table 2). Although several associations reached statistical significance in this large cohort, effect sizes were small, with HRs near 1.00, indicating modest absolute differences in treatment timing.

**Table 1.** Characteristics of Patients With Early-Onset Colorectal Cancer by 4-Level Rurality Classification

Characteristic	Patients, No (%)					*P* value
	Total (N = 79 090)	All urban (n = 52 611 [66.5%])	Mostly urban (n = 16 259 [20.6%])	Mostly rural (n = 5567 [7.0%])	All rural (n = 4653 [5.9%])	
Time to treatment, d						
Mean (SD)	20.0 (32.4)	20.7 (33.2)	19.1 (32.1)	17.8 (27.7)	18.3 (29.7)	<.001
Median (IQR)	0 (0-30)	0 (0-30)	0 (0-30)	0 (0-30)	0 (0-30)	
Missing	11 312 (14.3)	8541 (16.2)	1405 (8.6)	798 (14.3)	568 (12.2)_	
Sex						
Male	42 092 (53.22)	27 831 (52.9)	8668 (53.3)	3013 (54.1)	2580 (55.5)	.004
Female	36 998 (46.78)	24 780 (47.1)	7591 (46.7)	2554 (45.9)	2073 (44.5)	
Race						
American Indian or Alaska Native	510 (0.6)	231 (0.4)	140 (0.7)	64 (1.2)	75 (1.6)	<.001
Asian or Pacific Islander	7137 (9.0)	6177 (11.7)	838 (5.2)	81 (1.5)	41 (0.9)	
Black	10 915 (13.8)	8197 (15.6)	1998 (12.3)	411 (7.4)	309 (6.6)	
Hispanic	17 245 (21.0)	13 600 (25.9)	2847 (17.5)	488 (8.7)	310 (6.7)	
White	43 283 (54.7)	24 406 (46.4)	10 436 (64.2)	4523 (81.3)	3918 (84.2)	

**Table 2.** Association Between Sex, Race, and Timely Treatment Initiation Among Patients With Early-Onset Colorectal Cancer by 4-Level Rurality Classification

Treatment initiation time and characteristic	HR (95% CI)^a^			
	All urban	Mostly urban	Mostly rural	All rural
**30 d**				
Sex				
Female	1 [Reference]	1 [Reference]	1 [Reference]	1 [Reference]
Male	0.95 (0.93-0.97)	0.97 (0.97-1.00)	0.90 (0.85-0.96)	0.97 (0.91-1.05)
Race				
American Indian or Alaska Native	0.92 (0.79-1.08)	1.01 (0.84-1.22)	0.78 (0.58-1.06)	0.91 (0.69-1.21)
Asian or Pacific Islander	0.97 (0.93-1.00)	0.98 (0.90-1.06)	0.99 (0.76-1.29)	0.66 (0.42-1.02)
Black	0.96 (0.93-0.99)	1.06 (1.00-1.12)	1.19 (1.06-1.34)	1.09 (0.95-1.25)
Hispanic	0.94 (0.91-0.97)	0.94 (0.90-1.06)	1.09 (0.97-1.22)	1.00 (0.87-1.15)
White	1 [Reference]	1 [Reference]	1 [Reference]	1 [Reference]
60 d				
Sex				
Female	1 [Reference]	1 [Reference]	1 [Reference]	1 [Reference]
Male	0.95 (0.93-0.97)	0.97 (0.94-1.01)	0.91 (0.85-0.96)	0.98 (0.91-1.05)
Race				
American Indian or Alaska Native	0.90 (0.78-1.04)	0.96 (0.80-1.15)	0.76 (0.58-1.00)	0.91 (0.71-1.18)
Asian or Pacific Islander	0.96 (0.93-0.99)	0.95 (0.99-1.10)	0.94 (0.73-1.21)	0.79 (0.56-1.11)
Black	0.95 (0.93-0.98)	1.04 (0.99-1.10)	1.16 (1.03-1.30)	1.04 (0.92-1.18)
Hispanic	0.93 (0.91-0.96)	0.93 (0.88-0.97)	1.04 (0.93-1.16)	1.00 (0.88-1.13)
White	1 [Reference]	1 [Reference]	1 [Reference]	1 [Reference]
90 d				
Sex				
Female	1 [Reference]	1 [Reference]	1 [Reference]	1 [Reference]
Male	0.95 (0.93-0.97)	0.97 (0.94-1.01)	0.90 (0.85-0.96)	0.97 (0.91-1.04)
Race				
American Indian or Alaska Native	0.89 (0.78-1.02)	0.98 (0.82-1.16)	0.77 (0.59-1.00)	0.89 (0.70-1.15)
Asian or Pacific Islander	0.96 (0.93-0.99)	0.94 (0.87-1.01)	0.92 (0.72-1.17)	0.80 (0.57-1.11)
Black	0.95 (0.92-0.98)	1.04 (0.99-1.10)	1.15 (1.02-1.28)	1.03 (0.91-1.17)
Hispanic	0.93 (0.91- 0.95)	0.92 (0.88-0.97)	1.03 (0.93-1.15)	0.99 (0.87-1.12)
White	1 [Reference]	1 [Reference]	1 [Reference]	1 [Reference]

Abbreviation: HR, hazard ratio.

^a^ All models were adjusted for sociodemographic characteristics (age at diagnosis, marital status, and census tract socioeconomic level), tumor characteristics (stage, grade, and primary site), and year of diagnosis. Results from complete case analysis were consistent with those from the imputed models for the 90-day outcome. Among individuals residing in urban areas, male patients and those from racially and ethnically minoritized groups were less likely to receive treatment within 90 days.

## Discussion

Our cross-sectional analysis found that delays in treatment initiation—often exceeding 90 days—were more common in all-urban populations and appeared to disproportionately affect young male, Asian or Pacific Islander, Black, or Hispanic patients. Although absolute differences in average treatment timing were modest, our focus on clinically relevant delay thresholds (30, 60, and 90 days) is supported by recent meta-analytic literature.^2^ The consistency of these delays across sociodemographic groups challenges assumptions of uniformly timely access in urban settings. Overcrowded urban health care systems or inefficient public transportation may limit access to care.^4,5^ Additionally, young adults face distinct challenges across life stages, including lack of health insurance among patients aged 18 to 29 years and financial strain among patients aged 30 to 39 years that hinder timely access to treatment.^6^

Finally, this study focused on patterns in TTT rather than clinical impact, reflecting limitations related to the SEER population-based design and lack of day-level treatment timing data. Our findings also underscore the distinction between statistical significance and clinical importance: although several associations reached statistical significance in this large cohort, the observed HRs were small. Nonetheless, even modest delays may accumulate meaningful population-level disparities when they persist across sociodemographic groups, warranting additional investigation with more granular treatment timing and clinical data.

## Notes

Supplement 1. eMethods. Measures eReferences.

Supplement 2. Data Sharing Statement
