# Supplementary material for: Geographic, Racial, and Sex Disparities in Time to Treatment for Early-Onset Colorectal Cancer
Source: JAMA Netw Open. 2026 Mar 16;9(3):e261980. doi: 10.1001/jamanetworkopen.2026.1980 (PMC12993694; doi:10.1001/jamanetworkopen.2026.1980)
Supplement: Supplement 1. — eMethods. Measures eReferences. [file jamanetwopen-e261980-s001.pdf]

Supplemental Online Content

Tsai M, Coughlin SS, Cortes J, Vega KJ. Race, sex, and rurality in time to treatment for early-onset colorectal cancer. *JAMA Netw Open.* 2026;9(3):e261980.
doi:10.1001/jamanetworkopen.2026.1980

eTable. Measures

eReferences.

This supplemental material has been provided by the authors to give readers additional information about their work.

© 2026 Tsai M et al. *JAMA Network Open*.

eTable. Measures

Rurality

Rurality was classified using the Urban-Rural Indicator Code based on the percentage of residents in non-urban areas: all urban (100%), mostly urban (≥50%–<100%), mostly rural (>0%–<50%), and all rural (100%).

Time to Treatment

In the absence of a standardized definition of treatment timeliness in the literature, we adopted this categorization based on evidence showing increased mortality risk when treatment is delayed beyond four weeks[1]. Because SEER does not capture all treatment timing and modalities, some individuals may be misclassified if their first-line treatment was not recorded. Additionally, SEER reports time to treatment in months only, without providing exact dates of diagnosis or treatment; therefore, we converted months to days to align with existing literature[1]. These limitations are documented in SEER resources[2]. Nevertheless, SEER remains a widely used and reliable source for population-based cancer research, and its treatment information is among the most comprehensive available for large-scale analyses, particularly for studying disparities. To ensure transparency, we have explicitly noted this limitation in the manuscript's limitations section.

eReferences

1. Ungvari Z, Fekete M, Fekete JT, et al. Treatment delay significantly increases mortality in colorectal cancer: a meta-analysis. *Geroscience*. Jun 2025;47(3):5337-5353. doi:10.1007/s11357-025-01648-z

2. SEER Acknowledgment of Treatment Data Limitations, Surveillance, Epidemiology, and End Results Program, available from https://seer.cancer.gov/data-software/documentation/seerstat/nov2024/treatment-limitations-nov2024.html. Accessed on Nov 25, 2025.

© 2026 Tsai M et al. *JAMA Network Open*.
