# Supplementary material for: Geographic, Racial, and Sex Disparities in Time to Treatment for Early-Onset Colorectal Cancer
Source: JAMA Netw Open. 2026 Mar 16;9(3):e261980. doi: 10.1001/jamanetworkopen.2026.1980 (PMC12993694; doi:10.1001/jamanetworkopen.2026.1980)
Supplement: Supplement 2. — Data Sharing Statement [file jamanetwopen-e261980-s002.pdf]

Data Sharing Statement

Tsai. Geographic, Racial, and Sex Disparities in Time to Treatment for Early-Onset Colorectal Cancer. *JAMA Netw Open*. Published March 16, 2026.
doi:10.1001/jamanetworkopen.2026.1980

Data
Data available: Yes
Data types: Other (please specify)
Additional Information: The datasets generated during the current study are available in the Surveillance, Epidemiology, and End Results Program (https://seer.cancer.gov/) repository.
How to access data: The datasets generated during the current study are available in the Surveillance, Epidemiology, and End Results Program (https://seer.cancer.gov/) repository.
When available: With publication

Supporting Documents
Document types: None

Additional Information
Who can access the data: anyone requesting the data
Types of analyses: for a specified purpose
Mechanisms of data availability: without investigator support
